# Pollution Characteristics, Source Apportionment, and Health Risk Assessment of Potentially Toxic Elements (PTEs) in Road Dust Samples in Jiayuguan, Hexi Corridor, China

**DOI:** 10.3390/toxics10100580

**Published:** 2022-09-30

**Authors:** Kai Xiao, Xiaoqing Yao, Xi Zhang, Ning Fu, Qiuhong Shi, Xiaorui Meng, Xuechang Ren

**Affiliations:** 1 College of Environmental and Municipal Engineering, Lanzhou Jiaotong University, Lanzhou 730070, China; 2 Analysis and Testing Center, Gansu Province Environmental Monitoring Center, Lanzhou 730020, China

**Keywords:** Jiayuguan, road dust, potentially toxic elements, pollution characteristics, source apportionment, health risk assessment

## Abstract

The sources of potentially toxic elements (PTEs) in road dust are complex and potentially harmful to humans, especially in industrial cities. Jiayuguan is the largest steel-producing city in Northwest China, and this study was the first to conduct a related study on PTEs in road dust in this city, including the pollution characteristics, source apportionment, and health risk assessment of PTEs in road dust. The results showed that the highest concentration of PTEs in the local road dust samples were Mn, Ba, Zn, and Cr. The enrichment factor (EF) of Se was the highest, and it was “Very high enrichment” in areas other than the background area, indicating that the local Se was more affected by human activities. The geoaccumulation index (I_geo_) of Se was also the highest, and the pollution level was 5 in all areas except the background area, indicating that the local Se was more polluted and related to coal combustion. The sources of PTEs in local road dust samples mainly included geogenic-industrial sources, coal combustion, traffic sources, and oil combustion. For the non-carcinogenic risk, the hazard index (HI) of each element of children was higher than that of adults, and the sum of the HI of each element was greater than 1, indicating that there was a non-carcinogenic risk under the combined influence of multiple elements, which was especially obvious in industrial areas. For the carcinogenic risk, the cancer risk (CR) of Cr at a certain point in the industrial area exceeded 10^−4^, which was a carcinogenic risk, and the Cr in this area may be related to the topsoil of the local abandoned chromate plant.

## 1. Introduction

Since human civilization entered the 21st century, with the activities and development of industry, the pollutants produced by human activities have increased day by day, which has brought certain challenges to the living environment and health of human beings. Inhalable particulate matter (PM_10_) is particulate matter with a particle size of less than 10 μm in the air, and consists of organic and inorganic (trace metals, cations, and anions) species [1]. Long-term exposure to high concentrations of PM_10_ can cause certain harm to the human body, such as increasing the incidence of tuberculosis and causing circulatory diseases [2,3].

Road dust is composed of a mixture of particulate matter from various natural and man-made sources and contains certain harmful materials. It is one of the sources of PM_10_ and one of the important pollutants that causes urban environmental pollution [4,5,6]. The source of road dust is complex, and it is the carrier of various pollutants, and human activities have a great impact on the components of road dust. For example, under stable conditions, metallurgical dust, vehicle exhaust (non-exhaust particulate components), coal dust, and other particulate pollutants are mixed into road dust through gravity sedimentation and become a part of it, which leads to complex chemical components of road dust, including various ions, heavy metals and organics, etc. [7,8]. However, under the action of motor vehicles, pedestrians, and wind, low particle size road dust is usually suspended in the air again, causing the secondary pollution of particulate matter [9,10]. Therefore, the problem of road dust pollution has attracted public attention and has gradually become a research hotspot in urban pollution problems.

Potentially toxic elements (PTEs) have certain potential toxicity and can persist in dust, water, soil, and other media. Although some PTEs are beneficial to the organism at low concentrations and are essential trace elements, they are toxic when the concentration exceeds the tolerated dose of the organism, such as Cu, Zn, and Se [11,12]. Relevant studies have shown that PTEs in road dust have negative effects on human health, such as causing poisoning, cancer, respiratory diseases, and even psychological diseases [13,14,15]. In the road dust in different areas of the city, the content of PTEs usually shows a certain spatial difference due to the geographical location [16,17], which makes the impact of urban road dust on human health particularly complex. In the past few years, many scholars have done a lot of related research on PTEs in road dust, such as source apportionment, influencing factors, health risk assessment, and component characteristics [18,19,20]. The research sites of these studies were mostly in major cities in the world, but there were a few relevant research cases in the desert areas of northwest China, especially for industrial cities in the desert areas of northwest China, and a few reports have been reported in recent years.

Jiayuguan (98°17′ E, 39°47′ N) is located in the Hexi Corridor of Gansu Province, China, and is the largest steel production base in Northwest China. The local industry is dominated by metallurgical industry and material processing industry, supplemented by tourism. As one of the important industrial cities in the northwest China, road dust in this region is greatly affected by industrial emissions. According to the results of field investigation in this area, there were many chemical enterprises in Jiayuguan a few years ago, such as chromate plants and cement plants. Although these factories have been shut down one after another in recent years, the topsoil of the waste factories still contains a large amount of PTEs [21], which undoubtedly has a certain impact on the concentration of PTEs in the local road dust, and poses potential harm to the health of local residents. However, there are no relevant research reports on road dust in Jiayuguan, especially for PTEs in road dust in this area. For industrial cities, research on PTEs in road dust is of great significance. Such research can not only provide a scientific basis for local pollution prevention and control, but also provide a reference for local health departments to regulate relevant policies to ensure public health. Therefore, based on the above situation, the purpose of this study is as follows: (1) To collect road dust samples (16 samples in total) in each functional area and measure the concentration of PTEs to analyze the pollution characteristics of PTEs in road dust; (2) To determine the pollution levels and identifying sources of PTEs in road dust; and (3) Assess the health risks of PTEs in road dust.

## 2. Materials and Methods

### 2.1. Collection of Road Dust Samples and Chemical Analysis of PTEs

**Figure 1:**
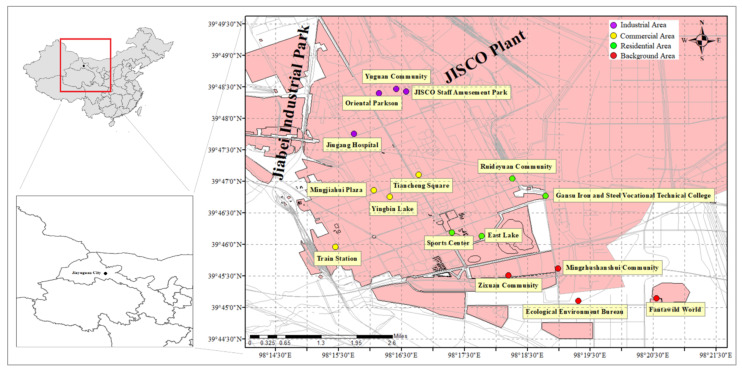
Distribution of sampling points.

Related studies in recent years showed that the urbanization rate of Jiayuguan was extremely high, exceeding 90% [22]. Therefore, this study focused on road dust in the urban area of Jiayuguan. In this study, the urban area was divided into industrial area, commercial area, residential area, and background area. Among the four functional areas, four sampling points were set up near the main road in each area, and the distribution of sampling points is shown in Figure 1. For the industrial area, the vehicles in this area were mainly large diesel vehicles, and the sampling point was close to Jiabei Industrial Park and “Jiuquan Iron & Steel (Group) Co., Ltd. (JISCO)” (Gansu, China), the largest iron and steel producer in the northwest China. For the commercial area, the vehicles in this area were mainly private cars, taxis, and buses, with a high traffic flow, and there were many shopping malls and various stores near the sampling point. For the residential area, the vehicles in this area were mainly private cars and buses, and there were many apartments and residential houses near the sampling point. For the background area, the area was far from the city center and was less affected by human activities.

In the winter of 2020, road dust samples were collected on the main roads around each sampling point. During sampling, brushes and dustpans were used to collect road dust samples, and 4–6 samples were collected around each sampling point and mixed. The mass of each sample was greater than 100 g. After the sample collection was completed, the samples were sealed with polyethylene bags, and the samples were promptly transferred to the laboratory to dry naturally. Then, 20-mesh (0.85-mm) and 100-mesh (0.15-mm) nylon sieves were used in sequence to remove the broken leaves, hair, stones, and other parts that are difficult to resuspend in the road dust. After the sample preparation was completed, the samples were stored under 4 °C and protected from light before chemical analysis.

Referring to the research results of other scholars [17,23], this study selected 12 PTEs (V, Cr, Mn, Co, Ni, Cu, Zn, As, Se, Cd, Ba, and Pb) with high pollution potential for chemical analysis. The sample digestion method is as follows: (1) Put 0.1 g of the sieved sample into a 50 mL Teflon crucible (Dechuang, Huizhou, China), add 5 mL of HNO_3_ (Aladdin, Shanghai, China), and digest on an electric hot plate (Keheng, Shanghai, China) at 120 °C for 5 min. (2) Add 2 mL H_2_O_2_ (Damao, Tianjin, China) to the crucible, raise the temperature to 160 °C for digestion for 5 min. (3) Add 2 mL of HF (Aladdin, Shanghai, China) to the crucible, raise the temperature to 180 °C for digestion for 5 min. (4) Remove the Teflon crucible from the electric hot plate, add 5 mL HNO_3_, 3 mL HF, 3 mL HClO_4_ (Xinyuan, Tianjin, China) in turn after cooling, cover the crucible, and digest on the electric hot plate at 180 °C for 1 h. (5) Remove the lid, wait for the solution to evaporate to nearly dryness, dilute it with ultrapure water in a 50 mL volumetric flask. After the samples were digested, the concentrations of 12 PTEs were measured by inductively coupled plasma mass spectrometer (ICP-MS) (7900, Agilent, Santa Clara, CA, USA). Strict quality control was implemented during the experiment. Blank samples, reference material of soil (GBW07446), and duplicate samples were used for quality assurance, and the pretreatment method was the same as that of road dust samples. In this study, no metal was measured in the blank sample, and the relative standard deviation of the duplicate samples was less than 2%. Information on the limits of detection and quantification is shown in Table S1.

### 2.2. Methods of Data Analysis

#### 2.2.1. Enrichment Factor (EF)

In the related studies of PTEs, Enrichment Factor (EF) is usually used to analyze the enrichment of PTEs and evaluate the impact of human activities on PTEs [24], and was calculated as follows:

(1) $$EF_{i}=\frac{{\left(C_{i}/C_{n}\right)}_{road\;dust}}{{\left(C_{i}/C_{n}\right)}_{soil}\;\;\;\;}$$

In the formula, *EF_i_* is the enrichment factor of element *i*; *C_i_* is the concentration of element *i* (mg·kg^−1^); *C_n_* is the concentration of reference element *n* (mg·kg^−1^). For the selection of reference elements, elements that have high concentration values in the environment and do not have any antagonistic and synergistic effects on the measured elements are usually selected, such as Mn, Al, Fe, and Sc [25,26]. Considering that the production process of the local metallurgical industry may contributed a certain amount of Fe and Al to the environment, Mn was selected as the reference element in this study. The background value of soil elements in Gansu Province [27] was selected as the concentration value of each element in the soil, and detailed information is shown in Table S2. The value of EF corresponds to the five pollution categories [28,29], as shown in Table 1.

#### 2.2.2. Index of Geoaccumulation (I_geo_)

An index of geoaccumulation is usually used to evaluate the pollution level of PTEs in an area [30]. It has been widely used in the study of sediment, soil, particulate matter, river, etc. [31,32,33]. The calculation formula is as follows:

(2) $$I_{geo}=\log_{2}\left(\frac{C_{n}}{k\times B_{n}}\right)$$

In the formula, *C_n_* is the concentration of element *n* in the sample (mg·kg^−1^); *B_n_* is the background value of element *n* in the soil (mg·kg^−1^); *k* is the correction coefficient, usually 1.5. The value of the geoaccumulation index corresponds to the six pollution categories [28], as shown in Table 2.

#### 2.2.3. Principal Component Analysis (PCA)

Principal component analysis (PCA) can reduce the dimensionality of the data, the principle of this method is to try to recombine the original variables into a new set of several comprehensive variables that are independent of each other, and according to actual needs, less comprehensive variables can be taken out to reflect as much information of the original variables as possible. At present, PCA is widely used in source apportionment studies of pollutants in various media, such as road dust, particulate matter, and soil [34,35]. In this study, PCA was used to determine the source of PTEs in road dust, and the SPSS (Statistical Product Service Solutions) software was used for the calculation of PCA.

#### 2.2.4. Health Risk Assessment

In this study, the EPA health risk assessment model was used to assess the health risk of PTEs in road dust [36]. The model can separately calculate the health risks of PTEs entering the human body through ingestion, inhalation, and dermal contact [37,38]. The average daily dose (ADD) for the three exposure pathways is calculated as follows:

(3) $$ADD_{ing}=\frac{c\times IngR\times EF\times ED\times CF}{BW\times AT}$$

(4) $$ADD_{inh}=\frac{c\times InhR\times EF\times ED}{PEF\times BW\times AT}$$

(5) $$ADD_{dermal}=\frac{c\times SA\times AF\times ABF\times EF\times ED\times CF}{BW\times AT}$$

In this formula, *ADD_ing_*, *ADD_inh_*, and *ADD_dermal_* are the average daily doses (mg·kg^−1^·d^−1^) of PTEs entering the human body through ingestion, inhalation, and dermal contact, respectively. The information of other parameters is shown in Table 3.

For the calculation of non-carcinogenic risk and carcinogenic risk, the calculation formula is as follows:

(6) $$HQ_{ij}=\frac{ADD_{ij}}{RfD_{i}}$$

(7) $$HI_{i}=\sum HQ_{ij}$$

(8) $$CR_{ij}=ADD_{ij}\times SF_{i}$$

(9) $$CR_{i}=\sum CR_{ij}$$

In these formulas, *i* is the PTE; *j* is the pathway of entry into the human body (ingestion, inhalation, dermal contact); *HQ* is the hazard quotient; *HI* is the hazard index; *CR* is the cancer risk; *RfD* is the reference dose of the PTE; *SF* is the slope factor. The information of *RfD* and *SF* is shown in Table 4. If *HI*<1, there is no obvious non-carcinogenic risk, and if *HI* > 1, there is a non-carcinogenic risk. If *CR* < 10^−6^, the carcinogenic risk is negligible, if the *CR* is between 10^−6^ and 10^−4^, the carcinogenic risk is at an acceptable level, and if *CR* > 10^−4^, there is a carcinogenic risk [39].

## 3. Results and Discussion

### 3.1. Concentration Characteristics of PTEs in Road Dust

**Figure 2:**
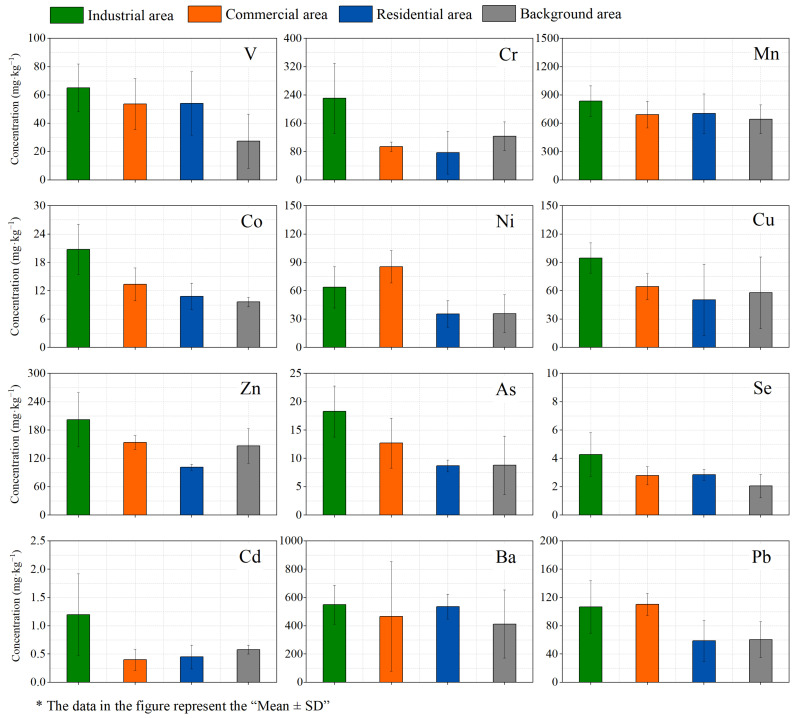
Concentration of PTEs in each area.

The concentration of PTEs in road dust samples in each area is shown in Figure 2. The mean total metal concentrations in the road dust samples decreased in the order: Mn > Ba > Zn > Cr > Pb > Cu > Ni > V > Co > As > Se > Cd. Mn is one of the elements widely distributed in the crust, and Cr Zn are mainly related to industrial sources. According to related research, the average content of Ba in Chinese coal (159 mg·kg^−1^) exceeded the world average (150 mg·kg^−1^), and the content of Ba in coal ash was about 4.2% [46], so coal combustion is one of the potential sources of Ba in the environment.

From the perspective of different areas, the concentration of PTEs in the industrial area was generally higher than that in the other three areas. The reason for this phenomenon was that there were a large number of metallurgical plants and chemical enterprises in the industrial area. These enterprises emitted a large amount of flue gas and particulate matter during the production process, and part of the flue gas and particulate matter settled to the surface under the action of gravity and mixed into the road dust, thereby increasing the concentration of PTEs in road dust in industrial areas, such as As and Se in coal combustion [47,48], and Cr, Cd, Cu, and Zn in metallurgy [49,50]. For the commercial area, the concentrations of Pb and Ni in the road dust in the commercial area were the highest among the four areas, and vehicle exhaust emissions were one of the potential sources of Pb and Ni [50,51]. Therefore, the phenomenon was caused by the emission of a large number of private cars and taxis in the commercial area. The concentrations of PTEs in road dust in residential area and background area were generally lower than those in industrial areas and commercial areas. Compared with the industrial area and the commercial area, the residential area and the background area are farther away from the industrial enterprises, with less traffic flow and less pollution.

### 3.2. Enrichment Characteristics of PTEs in Road Dust

**Figure 3:**
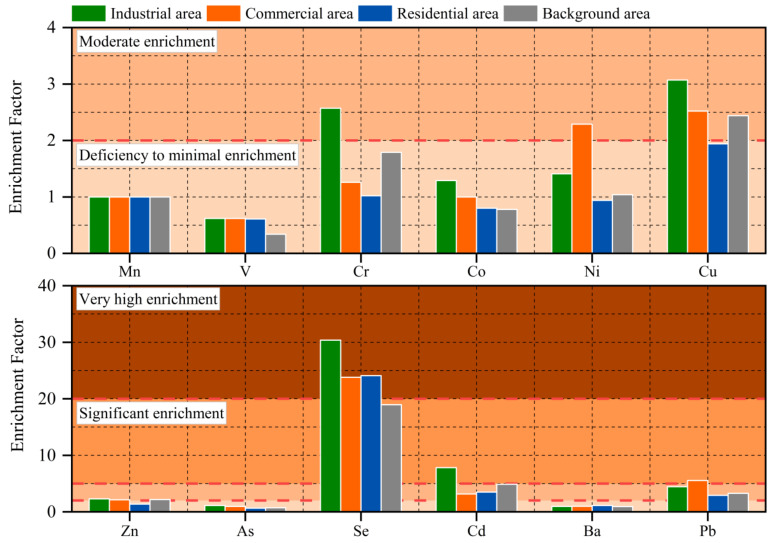
EF of PTEs in road dust in various areas.

The Enrichment Factor (EF) of PTEs in road dust samples is shown in Figure 3. From the EF value, the EF of Se was the highest, and it was more than 20 in all areas except the background area, which was “Very high enrichment”. The EF of Cd in the industrial area was greater than 5, which was “Significant enrichment”, and the EF in the other three areas was between 2 and 5, which was “Moderate enrichment”. The EF of Pb in the commercial area was greater than 5, which was “Significant enrichment”, and the EF in the other three areas was between 2 and 5, which was “Moderate enrichment”. Except for Se, Cd, and Pb, the EF of other PTEs in all areas were lower than 5, which were “Deficiency to minimal enrichment” or “Moderate enrichment” and were less affected by human activities.

From the perspective of various areas, the EF of Cr, Co, Cu, Zn, As, Se, and Cd in industrial areas were higher than those in other areas. The EF of Ni and Pb in commercial area were the highest, and the EF of these two elements were generally lower in other areas. According to the actual situation of each area, most of the industrial areas were metallurgical plants and chemical enterprises, and the commercial areas had high traffic flow. Therefore, compared to residential area and background area, industrial area and commercial area were more affected by human activities, especially vehicle exhaust emissions and the particulate matter emissions during industrial production and coal burning, which greatly increased the content of PTEs in road dust and increased the EF [52,53].

The EF of Se in road dust in Jiayuguan was the highest. Compared with Huainan [18] and Chengdu [54], which are both Chinese cities, the concentration of Se in road dust in Jiayuguan was higher than that in Huainan (1.200 mg·kg^−1^) and Chengdu (0.373 mg·kg^−1^), and the EF was much higher than that of Huainan (0.5), which is also an industrial city. Due to the large scale of steel production in Jiayuguan and the huge consumption of coal, a large amount of particulate matter was emitted in the process of burning coal, and part of it became part of the road dust through gravity sedimentation. In addition, unlike Huainan and Chengdu, Jiayuguan is located in northern China and is a typical northern city with a heating period. Therefore, in winter, the central heating of Jiayuguan increased the consumption of coal, and due to the limitation of the heating system, some villages and towns around the urban area could not obtain municipal heating supply. The residents of these villages and towns chose to burn coal for heating, which also increased the pollution caused by coal combustion and the EF of Se.

### 3.3. The Pollution Level of Each Area

**Figure 4:**
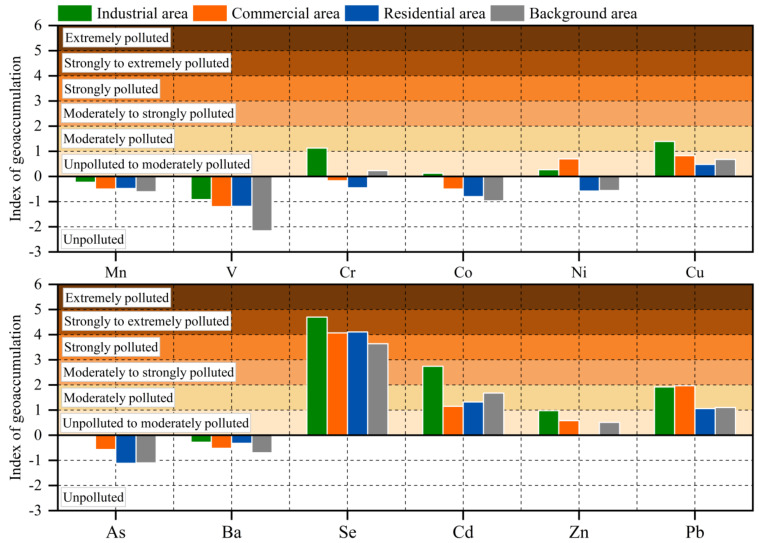
I_geo_ of PTEs in road dust in various areas.

The geoaccumulation index (I_geo_) of PTEs in road dust samples in various areas is shown in Figure 4. Among all PTEs, Se had the highest I_geo_ and was greater than 4 in all areas except the background area, and the pollution level was 5, which was “strong to extremely polluted”. The I_geo_ of Cd in the industrial area was between 2 and 3, and the pollution level was 3, which was “moderately to strongly pollution”. Except for Se and Cd, the I_geo_ of other PTEs in each area was less than 2, and the pollution level was below level 2. It is worth noting that the I_geo_ of Mn, V, As, and Ba were all less than 0, unpolluted. Similar to the enrichment factor, except for Ni and Pb, the I_geo_ of other PTEs in the industrial area was the highest in all areas, which indicated that the pollution in the industrial area was the most serious. For the commercial area, the I_geo_ of Ni and Pb in the commercial area was the highest, Ni and Pb mainly came from the exhaust emissions of motor vehicles [55,56], and could be mixed with the particulate matter into the road dust, so the result was in line with the characteristics of the large traffic flow in the commercial area.

According to the actual situation of industrial production in Jiayuguan, a large amount of coal was used in the production process of the local metallurgical industry, and the large amount of coal consumption increased the emission of elements such as Se and As [57,58]. Therefore, a large amount of particulate matter was emitted during the burning of coal, and this particulate matter contained a lot of Se. Additionally, the sedimentation to the surface increased the concentration of Se on the surface and increased the pollution of road dust. In addition, due to the limitation of the heating system, the residents of villages and towns around the Jiayuguan urban area used coal for heating in the winter, which also increased the emission of Se in the environment.

### 3.4. Source Apportionment of PTEs

Considering the limited number of samples in each area, in the process of principal component analysis, this study combined the sample data of four areas to determine the source of PTEs in road dust samples, and finally reflected the overall situation of road dust in Jiayuguan. The result is shown in Table 5. According to the result of PCA, a total of four components with eigenvalue greater than 1 were identified, and the cumulative variance contribution rate reached 74.67%.

PC1 explained 28.88% of the total variance, with higher loading for Cr, Co, and Cd. Although related research showed that Cr and Cd are related to emissions in metallurgical industrial production activities [17], some studies have shown that Co comes from iron ore and can be used as a tracer of particulate matter emitted by steel production [51]. However, according to the analysis results of the enrichment factor and geoaccumulation index in this study, the EF of Cr in all areas except industrial areas was less than 2, which was “Deficiency to minimal enrichment”, and only showed “Moderately polluted” in industrial areas. The EF of Co in all areas was less than 2, which was “Deficiency to minimal enrichment”, and studies have shown that Co is mainly from natural sources [59]. After the above analysis, the main source of Cr and Co in PC1 was considered to be a geogenic source. Therefore, the main source of PC1 was the geogenic source, followed by the industrial source.

PC2 explained 20.86% of the total variance, with higher loading for Mn, Ni, As, Se, and Pb. Among the elements with high loading, As and Se are representative elements of coal combustion [57,58]. Coal combustion is one of the sources of Mn in the environment. In addition to being related to vehicle exhaust emissions [55,56], Pb and Ni also come from the process of coal combustion [53,60]. Therefore, PC2 was identified as a coal combustion.

PC3 explained 13.35% of the total variance, with higher loading for Zn and As. Zn mainly comes from the wear of motor vehicle parts and exhaust emissions [1,53,61]. Usually, As is considered to be the representative element of coal combustion, but some studies have shown that As also comes from vehicle exhaust emissions [62]. Therefore, PC3 was identified as the traffic source.

PC4 explained 11.59% of the total variance, with higher loading for V and Ba. V is recognized as a tracer for oil combustion [63], and Ba is associated with diesel consumption emissions from diesel vehicles [64]. Therefore, PC4 was identified as oil combustion.

PTEs in road dust samples in Jiayuguan mainly came from geogenic-industrial sources, coal combustion, traffic sources, and oil combustion. The sources of road dust were complex, which was a collection of various particulate pollutants. According to the results of source apportionment, PTEs in road dust in Jiayuguan were mainly related to human activities. As the largest steel manufacturing city in Northwest China, the industrial production process of Jiayuguan was accompanied by huge consumption of coal and oil. Therefore, a large amount of particulate matter was emitted during industrial production and motor vehicle transportation. These particles contained a large amount of PTEs, and finally settled to the surface by gravity and mixed into the road dust.

### 3.5. Health Risk Assessment of PTEs in Road Dust

#### 3.5.1. Non-Carcinogenic Risk Assessment

The hazard quotient (HQ) for each category of ingestion is shown in Table 6. From the perspective of different groups, the HQ_ing_ and HQ_inh_ of children were higher than that of adults, and the HQ_dermal_ of adults was higher than that of children. This phenomenon is similar to the research results of some scholars [18,23,53]. Compared with adults, children have a weaker constitution, so they have lower resistance to PTEs pollution in the environment, especially for inhalation and ingestion. For the dermal contact route, the skin surface area (SA) and skin adherence factor (AF) of adult are higher than those of children. Due to the large difference in these parameters, and AF having a large influence on the intake of PTEs [65,66], the calculation result of HQ usually presents as “HQ_dermal_ (adult) > HQ_dermal_ (children)”. For each element, the HQ of each element for various exposure routes was less than 1, and there was no obvious non-carcinogenic risk for a single exposure route corresponding to a single element.

**Figure 5:**
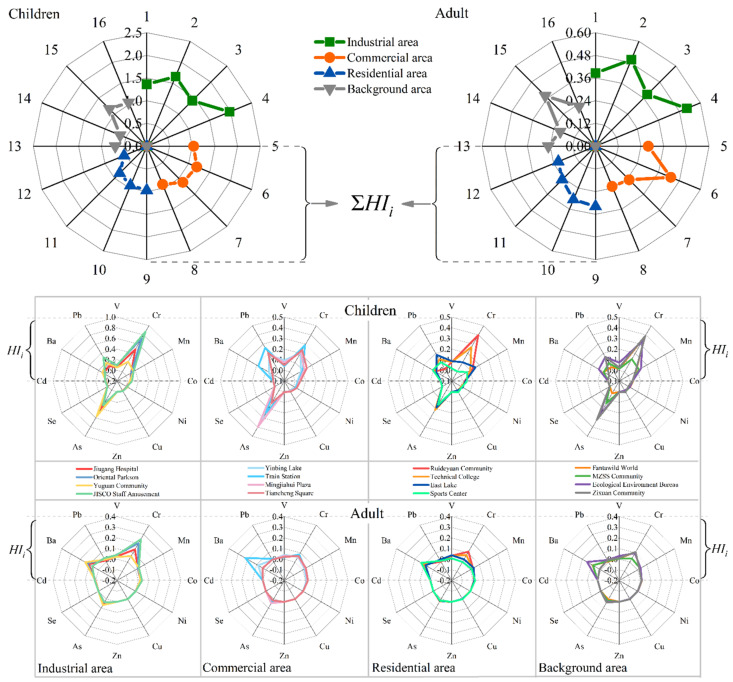
Characterization results for non-carcinogenic risk.

Figure 5 reflects the HI_i_ of each sampling point and the sum of the HI_i_ (∑HI_i_) of each area. For children, the non-carcinogenic risk of Cr and As was more obvious, the HI of Cr and As in industrial area was close to 1, and the non-carcinogenic risk was relatively high. The HI of each element for adults was lower than 1, and each element had no obvious non-carcinogenic risk. From the perspective of different areas, the HI of each element in the industrial area was relatively higher than other areas, which indirectly reflected the actual situation of serious pollution in the industrial area. The ∑HI_i_ of each element was calculated to reflect the comprehensive impact of all elements on the non-carcinogenic risk of each group. The ∑HI_i_ of adults in the four areas was less than 1, indicating no non-carcinogenic risk. For children, the ∑HI_i_ of all points in the industrial area was greater than 1, the ∑HI_i_ of three points in the commercial area was greater than 1, the ∑HIi of all points in the residential area was less than 1, and the ∑Hii of two points in the background area was greater than 1.

To sum up, the non-carcinogenic risk of PTEs in road dust in Jiayuguan was higher for children than for adults, and except for residential areas, PTEs, at some points in the rest of the area, present non-carcinogenic risks to local children. Therefore, it is suggested that the local environmental protection and health departments pay attention to the pollution of PTEs in road dust, especially the emission of PTEs in industrial areas, which should receive strengthened supervision.

#### 3.5.2. Carcinogenic Risk Assessment

The cancer risk (CR) for each category of intake is shown in Table 7. From the perspective of different groups, the CR_ing_ of children was greater than that of adults, and the CR_inh_ and CR_dermal_ of children were smaller than that of adults, which indicates that children are more susceptible to ingestion pathways than adult. The mean values of CR_ij_ in the four areas were all between 10^−4^ and 10^−6^, which indicated that the carcinogenic risk of each element under a single intake route was at an acceptable level.

The calculation results and related information of CR_i_ for children and adults in each area are shown in Table 8. For each element, the mean values of CR of Co and Cd in each area were lower than 10^−6^, and its carcinogenic risk was negligible. The mean values of CR of Cr, Ni, and As were between 10^−4^ and 10^−6^, and the carcinogenic risk was at an acceptable level. It is worth noting that in the industrial area, the maximum CR of Cr to children and adults reached 1.09 × 10^−4^ and 1.10 × 10^−4^, respectively, which exceeded 1 × 10^−4^, and there was a risk of cancer. Cr can accumulate in the human body and cause a series of gastrointestinal reactions before the lesions, and when too much Cr accumulates in the human body, it may cause lung cancer and gastric cancer [67]. According to the local field investigation results, there were some chromate plants in the “Jiabei industrial park” in the north of the city, and these chromate plants were discontinued a few years ago. The Cr in the topsoil of these historically abandoned factories seriously exceeded the standard, which presents potential harm to the health of the local population. At present, Cr in the topsoil of these abandoned factories has attracted the attention of local environmental protection departments, and soil remediation work has been carried out since 2020. In future research on the local environment, this work deserves continuous attention.

The mean CR of PTEs in Jiayuguan road dust samples was lower than 10^−4^, and the carcinogenic risk was at an acceptable level or low risk. However, the CR of Cr at individual point in the industrial area was higher than 10^−4^, and there was still a carcinogenic risk. A potential source of Cr in industrial area was topsoil from abandoned chromate plants. Therefore, it is recommended that the local environmental protection and health departments continue to pay attention to the soil remediation work in this area.

## 4. Conclusions

Based on the data of PTEs in Jiayuguan road dust samples, this study calculated the enrichment factor and geoaccumulation index of PTEs in Jiayuguan road dust, identified the source of PTEs by principal component analysis, and the health risk of PTEs to different local groups was assessed by the EPA health risk assessment model. The conclusions are as follows.

(1) Among the 12 PTEs (V, Cr, Mn, Co, Ni, Cu, Zn, As, Se, Cd, Ba, and Pb) in road dust samples, the highest concentrations were Mn, Ba, Zn, and Cr, and the concentration of PTEs in industrial areas was higher than the other three functional areas as a whole.

(2) For the EF of PTEs in road dust samples, the EF of Se was the highest, and the EF of Se in industrial area, commercial area, and residential area all exceeded 20, which was “very high enrichment”. The EF of Cd in the industrial area exceeded 5, which was “Significant enrichment”. The EF of Pb in the commercial area exceeded 5, which was “significant enrichment”. The EF of the remaining elements were low, with “Moderate enrichment” or “Deficiency to minimal enrichment “ in each area.

(3) The calculation of the I_geo_ showed that for most elements, the pollution level of the industrial area was higher than that of the rest area. For each element, the Igeo of Se was the highest, which was higher than 4 in all areas except the background area, and the pollution level was 5, which was “Strongly to extremely polluted”. The pollution level of Cd in the industrial area was 3, which was “Moderately to strongly polluted”. The pollution level of other elements in each area did not exceed level 2.

(4) According to the source apportionment results, the PTEs in Jiayuguan road dust mainly came from geogenic-industrial sources, coal combustion, traffic sources, and oil combustion.

(5) For non-carcinogenic risk, the HI of each element for children was higher than that for adults, and the sum of the HI of each element to children at some points in the industrial area, commercial area and background area exceeds 1, indicating a non-carcinogenic risk. From the perspective of different areas, the HI of the points in the industrial area was higher than that of the other areas, indicating that the non-carcinogenic risk of the industrial area was higher than that of the other areas.

(6) For the carcinogenic risk, the mean CR of each element in each region was less than 10^−4^, indicating that the carcinogenic risk was at an acceptable level or low risk. However, the CR at a certain point in the industrial area exceeded 10^−4^, indicating a carcinogenic risk, and the source of Cr in this area may be related to the topsoil of the local abandoned chromate plant.

According to the above conclusions, it is suggested that the local government should pay attention to coal-fired emissions and introduce relevant policies and regulations to reduce the pollution of Se to the environment, and focus on the health of children, especially the health effects of PTEs in industrial areas on children. In addition, the local government should continue to pay attention to the topsoil pollution of the abandoned chromate plant and take relevant measures to reduce the Cr pollution of the topsoil in the area.

## Figures and Tables

**Table 1:** The value of EF and the pollution category.

Value of EF	Pollution Category
(0, 2)	Deficiency to minimal enrichment
[2, 5)	Moderate enrichment
[5, 20)	Significant enrichment
[20, 40)	Very high enrichment
[40, +∞)	Extremely high enrichment

**Table 2:** The value of I_geo_ and the pollution category.

Value of I_geo_	Level	Pollution Category
[−∞, 0)	0	Unpolluted
[0, 1)	1	Unpolluted to moderately polluted
[1, 2)	2	Moderately polluted
[2, 3)	3	Moderately to strongly polluted
[3, 4)	4	Strongly polluted
[4, 5)	5	Strongly to extremely polluted
[5, +∞)	6	Extremely polluted

**Table 3:** Exposure parameters for health risk assessment model.

Parameter	Meaning	Unit	Children	Adult	Source
IngR	Ingestion rate	mg·d^−1^	200	100	[25,40,41,42,43,44]
InhR	Inhalation rate	mg·d^−1^	5	20	
EF	Exposure frequency	d·a^−1^	180	180	
ED	Exposure duration	a	6	24	
BW	Body weight	kg	15	70	
PEF	Particle emission factor	m^3^·kg^−1^	1.32 × 10^9^	1.32 × 10^9^	
AT (carcinogen)	Average time	d	365·70	365·70	
AT (non-carcinogen)	Average time	d	365·ED	365·ED	
SA	Skin surface area	cm^2^	2800	5700	
AF	Skin adherence factor	mg·(cm^2^·d)^−1^	0.2	0.7	
ABF	Absorption factor	/	0.001	0.001	
CF	Conversion factor	kg/mg	1 × 10^−6^	1 × 10^−6^	

**Table 4:** RfD and SF value of different elements for different exposure routes.

PTEs	RfDing	RfDinh	RfDdermal	SF_ing_	SF_inh_	SF_dermal_
V	7.00 × 10^−3^	1.40 × 10^−5^	7.00 × 10^−5^	-	-	-
Mn	4.66 × 10^−2^	1.43 × 10^−5^	1.84 × 10^−3^	-	-	-
Pb	3.50 × 10^−3^	3.50 × 10^−3^	5.25 × 10^−4^	-	-	-
Cu	3.70 × 10^−2^	4.00 × 10^−2^	1.20 × 10^−2^	-	-	-
Zn	3.00 × 10^−1^	3.00 × 10^−1^	6.00 × 10^−2^	-	-	-
Se	5.00 × 10^−3^	5.70 × 10^−5^	2.20 × 10^−3^	-	-	-
Ba	2.00 × 10^−1^	1.40 × 10^−2^	1.43 × 10^−4^			
Cd	1.00 × 10^−3^	1.00 × 10^−3^	1.00 × 10^−5^	0.38	6.30	3.00
Co	3.00 × 10^−3^	6.00 × 10^−5^	2.80 × 10^−5^	-	9.80	-
As	3.00 × 10^−4^	1.23 × 10^−4^	1.23 × 10^−4^	1.50	15.10	3.66
Cr	3.00 × 10^−3^	2.85 × 10^−5^	6.00 × 10^−5^	0.50	42.00	20.00
Ni	2.00 × 10^−2^	2.06 × 10^−2^	5.40 × 10^−3^	0.84	1.70	42.50
Source	[25,44,45]					

**Table 5:** Principal component analysis of overall road dust in Jiayuguan.

Elements	Principal Components			
	PC1	PC2	PC3	PC4
V	0.433	0.415	0.004	0.532
Cr	0.893	−0.076	0.197	0.154
Mn	0.300	0.714	−0.271	0.293
Co	0.765	0.344	0.185	−0.294
Ni	−0.147	0.850	0.182	−0.048
Cu	0.450	0.012	0.279	−0.764
Zn	0.057	−0.048	0.929	0.012
As	0.242	0.463	0.613	0.084
Se	0.694	0.558	−0.026	−0.170
Cd	0.903	0.124	−0.056	−0.142
Ba	−0.058	−0.041	0.302	0.512
Pb	0.469	0.657	0.098	−0.081
Eigenvalue	3.47	2.50	1.60	1.39
Variance contribution (%)	28.88	20.86	13.35	11.59
Cumulative variance contribution (%)	28.88	49.74	63.09	74.67

**Table 6:** HQ_ij_ for different intake route.

Elements	Children			Adult		
	HQ_ing_	HQ_inh_	HQ_dermal_	HQ_ing_	HQ_inh_	HQ_dermal_
V	4.70 × 10^−2^	4.45 × 10^−4^	1.31 × 10^−2^	5.03 × 10^−3^	3.81 × 10^−4^	2.01 × 10^−2^
Cr	2.88 × 10^−1^	5.73 × 10^−4^	4.03 × 10^−2^	3.08 × 10^−2^	4.92 × 10^−4^	6.15 × 10^−2^
Mn	1.01 × 10^−1^	6.25 × 10^−3^	7.19 × 10^−3^	1.09 × 10^−2^	5.36 × 10^−3^	1.10 × 10^−2^
Co	2.99 × 10^−2^	2.83 × 10^−5^	8.97 × 10^−3^	3.20 × 10^−3^	2.43 × 10^−5^	1.37 × 10^−2^
Ni	1.81 × 10^−2^	3.33 × 10^−7^	1.88 × 10^−4^	1.94 × 10^−3^	2.86 × 10^−7^	2.87 × 10^−4^
Cu	1.19 × 10^−2^	2.08 × 10^−7^	1.03 × 10^−4^	1.27 × 10^−3^	1.78 × 10^−7^	1.57 × 10^−4^
Zn	3.31 × 10^−3^	6.26 × 10^−8^	4.63 × 10^−5^	3.54 × 10^−4^	5.37 × 10^−8^	7.07 × 10^−5^
As	2.65 × 10^−1^	1.23 × 10^−5^	1.81 × 10^−3^	2.84 × 10^−2^	1.05 × 10^−5^	2.77 × 10^−3^
Se	3.93 × 10^−3^	6.53 × 10^−6^	2.50 × 10^−5^	4.21 × 10^−4^	5.59 × 10^−6^	3.82 × 10^−5^
Cd	4.32 × 10^−3^	8.17 × 10^−8^	1.21 × 10^−3^	4.62 × 10^−4^	7.00 × 10^−8^	1.84 × 10^−3^
Ba	1.62 × 10^−2^	4.37 × 10^−6^	6.33 × 10^−2^	1.73 × 10^−3^	3.75 × 10^−6^	9.66 × 10^−2^
Pb	1.58 × 10^−1^	2.99 × 10^−6^	2.95 × 10^−3^	1.69 × 10^−2^	2.57 × 10^−6^	4.50 × 10^−3^

**Table 7:** CR_ij_ for different intake route.

Elements	Children			Adult		
	CR_ing_	CR_inh_	CR_dermal_	CR_ing_	CR_inh_	CR_dermal_
Cr	3.70 × 10^−5^	5.88 × 10^−8^	4.14 × 10^−6^	1.59 × 10^−5^	2.02 × 10^−7^	2.53 × 10^−5^
Co	-	1.43 × 10^−9^	-	-	4.89 × 10^−9^	-
Ni	2.61 × 10^−5^	1.00 × 10^−9^	3.70 × 10^−6^	1.12 × 10^−5^	3.43 × 10^−9^	2.26 × 10^−5^
As	1.02 × 10^−5^	1.95 × 10^−9^	7.00 × 10^−8^	4.39 × 10^−6^	6.69 × 10^−9^	4.27 × 10^−7^
Cd	1.41 × 10^−7^	4.41 × 10^−11^	3.11 × 10^−9^	6.02 × 10^−8^	1.51 × 10^−10^	1.90 × 10^−8^

**Table 8:** CR_i_ for children and adults in various areas.

Area		Children					Adult				
		Cr	Co	Ni	As	Cd	Cr	Co	Ni	As	Cd
Industrial area	Max	1.09 × 10^−4^	3.07 × 10^−9^	5.09 × 10^−5^	2.17 × 10^−5^	5.26 × 10^−7^	1.10 × 10^−4^	1.05 × 10^−8^	5.77 × 10^−5^	1.02 × 10^−5^	2.90 × 10^−7^
	Min	2.76 × 10^−5^	1.58 × 10^−9^	1.90 × 10^−5^	1.20 × 10^−5^	1.09 × 10^−7^	2.77 × 10^−5^	5.42 × 10^−9^	2.16 × 10^−5^	5.61 × 10^−6^	6.05 × 10^−8^
	Mean	7.23 × 10^−5^	2.17 × 10^−9^	3.44 × 10^−5^	1.56 × 10^−5^	2.63 × 10^−7^	7.26 × 10^−5^	7.44 × 10^−9^	3.90 × 10^−5^	7.28 × 10^−6^	1.45 × 10^−7^
Commercial area	Max	3.62 × 10^−5^	1.88 × 10^−9^	5.54 × 10^−5^	1.53 × 10^−5^	1.31 × 10^−7^	3.64 × 10^−5^	6.46 × 10^−9^	6.28 × 10^−5^	7.17 × 10^−6^	7.26 × 10^−8^
	Min	2.53 × 10^−5^	8.79 × 10^−10^	3.51 × 10^−5^	5.45 × 10^−6^	2.19 × 10^−8^	2.54 × 10^−5^	3.01 × 10^−9^	3.98 × 10^−5^	2.55 × 10^−6^	1.21 × 10^−8^
	Mean	2.94 × 10^−5^	1.40 × 10^−9^	4.62 × 10^−5^	1.08 × 10^−5^	8.76 × 10^−8^	2.96 × 10^−5^	4.79 × 10^−9^	5.24 × 10^−5^	5.06 × 10^−6^	4.84 × 10^−8^
Residential area	Max	4.97 × 10^−5^	1.61 × 10^−9^	2.93 × 10^−5^	8.26 × 10^−6^	1.31 × 10^−7^	4.99 × 10^−5^	5.52 × 10^−9^	3.32 × 10^−5^	3.86 × 10^−6^	7.26 × 10^−8^
	Min	4.71 × 10^−7^	8.26 × 10^−10^	8.43 × 10^−6^	6.04 × 10^−6^	2.19 × 10^−8^	4.73 × 10^−7^	2.83 × 10^−9^	9.56 × 10^−6^	2.83 × 10^−6^	1.21 × 10^−8^
	Mean	2.41 × 10^−5^	1.13 × 10^−9^	1.91 × 10^−5^	7.39 × 10^−6^	9.85 × 10^−8^	2.42 × 10^−5^	3.88 × 10^−9^	2.17 × 10^−5^	3.45 × 10^−6^	5.44 × 10^−8^
Background area	Max	4.86 × 10^−5^	1.17 × 10^−9^	2.78 × 10^−5^	1.24 × 10^−5^	1.53 × 10^−7^	4.88 × 10^−5^	4.02 × 10^−9^	3.15 × 10^−5^	5.81 × 10^−6^	8.47 × 10^−8^
	Min	1.72 × 10^−5^	8.89 × 10^−10^	8.11 × 10^−7^	1.28 × 10^−6^	1.09 × 10^−7^	1.73 × 10^−5^	3.05 × 10^−9^	9.19 × 10^−7^	5.97 × 10^−7^	6.05 × 10^−8^
	Mean	3.88 × 10^−5^	1.01 × 10^−9^	1.94 × 10^−5^	7.49 × 10^−6^	1.26 × 10^−7^	3.90 × 10^−5^	3.45 × 10^−9^	2.20 × 10^−5^	3.50 × 10^−6^	6.95 × 10^−8^

## Data Availability

All data included in this study are available upon request by contact with the corresponding author.

## Supplementary Materials

The following are available online at https://www.mdpi.com/article/10.3390/toxics10100580/s1, Table S1: Detection limit and quantification limit, Table S2: Background values of soil elements in Gansu Province, China.
