# Supplementary material for: Pollution Characteristics, Source Apportionment, and Health Risk Assessment of Potentially Toxic Elements (PTEs) in Road Dust Samples in Jiayuguan, Hexi Corridor, China
Source: Toxics. 2022 Sep 30;10(10):580. doi: 10.3390/toxics10100580 (PMC9607028; doi:10.3390/toxics10100580)
Supplement: Supplementary file 1 [file toxics-10-00580-s001.zip › toxics-1899132-supplementary.pdf]

Table S1 Detection limit and quantification limit

Element	Limit of Detection (mg·kg^{-1})	Limit of Quantitation (mg·kg^{-1})
V	0.7	2.8
Cd	0.07	0.28
Mn	0.7	2.8
Co	0.02	0.07
Ni	0.6	2
Cu	0.5	2.0
Zn	7	28
As	0.6	2.4
Se	0.4	1.6
Cr	2	8
Ba	1.1	4
Pb	0.5	2

Table S2 Background values of soil elements in Gansu Province, China

Element	Elemental Background Values of Soil (mg·kg^{-1})
V	81.9
Cr	70.2
Mn	653
Co	12.6
Ni	35.2
Cu	24.1
Zn	68.5
As	12.6
Se	0.11
Cd	0.12
Ba	446
Pb	18.8
